# A procurement‐based classification of pharmaceutical supplies for diabetes disease management

**DOI:** 10.1002/hsr2.807

**Published:** 2022-09-14

**Authors:** Yousef Abdulsalam, Abdullah Alibrahim, Dari Alhuwail, Hashem Behbehani

**Affiliations:** ^1^ Department of Information Systems and Operations Management, College of Business Administration Kuwait University Kuwait City Kuwait; ^2^ Department of Industrial and Management Systems Engineering, College of Engineering and Petroleum Kuwait University Kuwait City Kuwait; ^3^ GeoHealth Lab Dasman Diabetes Institute Kuwait City Kuwait; ^4^ Department of Information Science, College of Life Sciences Kuwait University Kuwait City Kuwait; ^5^ Health Informatics Unit Dasman Diabetes Institute Kuwait City Kuwait

**Keywords:** diabetes care, inventory, pharmaceutical supplies, procurement, supply management

## Abstract

**Background and Aims:**

Diabetes is among the most prevalent noncommunicable chronic diseases globally and carries a substantial expense in worldwide health care. Pharmaceutical supplies related to diabetes management account for 20%–40% of the disease's management cos, and this percentage continues to increase. This study examines the pharmaceutical expenses associated with one of the most common chronic diseases: diabetes. Specifically, we measure the extent to which patient health and demographic factors drive the annual cost of pharmaceutical supplies for diabetes management. Second, the study applied a procurement‐centric classification scheme to pharmaceutical items involved in diabetes treatment.

**Methods:**

Data on 98,648 pharmaceutical‐dispensing transactions (related to 2828 patients) over 1 year were collected from a specialized diabetes health center. Pharmaceutical prices from the sample were compared internationally to ensure that the findings apply to other countries. The association between the item cost and the number of unique patients prescribed pharmaceutical products was estimated at the category and subcategory levels.

**Results:**

Approximately 80% of total pharmaceutical expenditures were attributed to 20% of patients. Two of 20 pharmaceutical categories—anti‐diabetes drugs and insulin—accounted for 34% of products dispensed and 57% of total pharmaceutical expenditures. Age, body mass index, and diabetes type were essential factors in predicting supply cost per patient.

**Conclusion:**

Applying the portfolio purchasing model also suggested that some clinically similar items, like insulin types, are best procured through divergent procurement strategies or vendors for optimal cost efficiency. A better understanding of the diverse array of diabetes supplies can reveal opportunities for better strategic supply management. This supply classification approach can also be applied in other supply‐intensive specialties, such as orthopedics.

## 1 INTRODUCTION

Pharmaceutical supplies are integral to delivering health care, particularly for chronic diseases such as diabetes, which require ongoing follow‐up and management.^1^ An estimated 75% of total health spending goes toward managing chronic diseases.^2^, ^3^ Although many studies have considered the economic cost of treating chronic noncommunicable diseases,^4^, ^5^ fewer studies focused on pharmaceutical costs. Supply costs represent the second largest category at hospitals, after labor, accounting for 15%–25% of total hospital costs.^6^ Even though supply costs are less than labor costs in healthcare delivery, much research has identified significant savings in supply expenditures without compromising clinical performance. In addition to cost‐saving strategies that materials managers can apply, studies also showed substantial cost‐efficiency gains could be achieved through better physician awareness and patient education.^2^, ^7^, ^8^

This study focused on the cost of pharmaceutical supplies used for diabetes management. Diabetes is one of the most prevalent chronic diseases globally, affecting about 8% of the world's population.^9^ More research about the pharmaceutical costs related to this disease is warranted because of the disease's prevalence and because medications dispensed to people with diabetes cost more than twice as much as those without diabetes.^10^ Most dispensed pharmaceuticals are insulin and anti‐diabetes drugs. Nondrug items include needles, ointments, and other commodities. The pharmaceutical costs per patient per year are consistent across different countries, compared to hospital costs or additional indirect costs. Therefore, insight gained from examining this cost category can be more readily generalized to other health facilities, chronic conditions, and countries.

Building on previous diabetes cost research, which is briefly reviewed in the subsequent section, this study aimed to achieve two research objectives related to pharmaceutical costs. The first objective was to analyze the proliferation and costs of pharmaceuticals related to diabetes disease management. The subsequent objective was to understand the nature of the dispensed pharmaceutical products from a procurement perspective. Adapting the portfolio purchasing model^11^ provided a classification scheme for diverse supplies related to diabetes treatment based on their financial and risk impacts. These objectives were achieved by analyzing data from a tertiary diabetes treatment center with about 2800 patients who received 98,000 pharmaceutical items in 1 year.

## 2 BACKGROUND

### 2.1 The global prevalence of diabetes

Diabetes mellitus is a chronic noncommunicable disease in which the body cannot produce or properly use insulin, a hormone that regulates the amount of glucose in the blood. A 2021 report by the International Diabetes Federation estimated that the disease affected over 500 million of the world's population between 20 and 79 years.^9^ Diabetes is among the most prevalent conditions globally and accounts for 11% of health care expenditures worldwide.^9^, ^12^ This number is supported by more recent studies in high median‐income countries: 10% in Germany,^10^ 8.3% in Italy,^13^ and 14% in the United States.^14^ These estimates may even be conservative because some researchers have estimated that more than 40% of people with diabetes worldwide are undiagnosed.^4^

Sun, Hong, et al. “IDF Diabetes Atlas: Global, regional and country‐level diabetes prevalence estimates for 2021 and projections for 2045.” *Diabetes research and clinical practice* 183 (2022): 109119.

Although the incidence of death directly related to diabetes has significantly dropped in the last 30 years, the number of patients with diabetes continues to increase.^15^ For example, the number of adults reporting a diabetes diagnosis in the United States tripled between 1990 and 2010, from 6.5 million to 20.7 million, whereas the population only increased by 27%.^16^ Similarly, the number of people with diabetes in the United Kingdom has doubled in 20 years.^17^ Forecasts suggest that this trend will continue, with the number of patients with type 1 and type 2 diabetes expected to increase by more than 50% between 2015 and 2030.^18^

### 2.2 Cost of diabetes

A systematic literature review of 75 studies found that the direct annual treatment cost of diabetes ranged from USD 966 billion worldwide, which factors to USD 1700 per diabetic patient.^9^ Direct costs related to diabetes consist of inpatient hospital visits, medication, laboratory, and equipment costs. Medication costs for patients with diabetes are 2.2 times higher than medication costs for people without diabetes, and the inpatient hospital cost is 1.8 times higher for patients with diabetes.^10^ A similar study in Spain revealed that overall treatment costs were 74% higher for patients with diabetes than those without diabetes when controlling for age and gender. Medication costs were 89% higher for patients with diabetes.^19^ Table 1 presents the ratios of medication costs to total direct costs from studies of similar scope in various developed countries. More exhaustive literature reviews of diabetes cost studies are available.^4^, ^9^ Medication costs account for 20%–40% of total direct treatment costs in developed countries.

**Table 1:** The ratio of medication costs to total direct costs per patient with diabetes (annual)

Country	Total direct cost per patient (€)	Medication costs per patient (€)	Ratio (%)
Germany	5899	1149	19
Italy	2756	814	30
Spain	1708	632	37
United Kingdom	5470	1153	21
France	5432	1458	27
Netherlands	3526	575	16
United States	8651	3905	45

*Note*: Germany, Italy, Spain, UK, and France data are based on 2010 estimates and adapted from Kanavos et al.^20^; the Netherlands data are based on 2016 data from Peters et al.,^21^ and United States data are based on 2017 data from the American Diabetes Association,^14^ applying a currency conversion rate of 1 EUR = 1.11 USD.

### 2.3 Supply categories and costs in diabetes management

Pharmaceutical costs related to diabetes treatment have been rising faster than overall treatment costs, warranting more attention to this cost category.^14^ In the United States, insulin costs tripled from 2002 to 2013.^22^ Then they nearly doubled from 2012 to 2016.^23^ Besides antidiabetes drugs and insulin, various medications and supplies are needed to manage diabetes and confounding diseases.

Diabetes medications differ dramatically in demand, price, criticality, and substitutability, posing a challenge to health administrators pursuing cost‐effective supply management. However, limited research has examined the costs and categories of various diabetes pharmaceutical supplies. A contribution of this study is its application of a structured classification scheme to the multiple categories of diabetes‐related pharmaceutical supplies to generate insights regarding more cost‐effective health services for diabetes. We approached this from a supply chain management perspective, a field dedicated to improving sourcing, supply selection, procurement, and inventory management.

**Figure 1:**
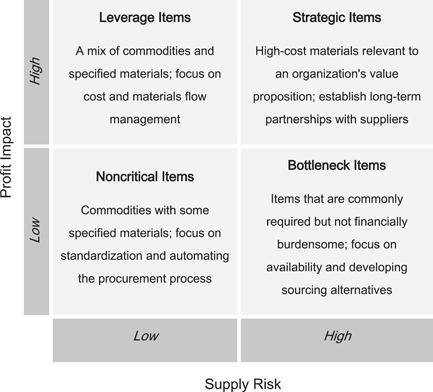
The portfolio purchasing model (adapted from Kraljic^11^).

The supply management literature contains numerous classification frameworks to stratify and effectively manage diverse supplies strategically.^24^, ^25^, ^26^ Among the most prolific of these frameworks is the purchasing portfolio model (Figure 1). Initially developed by Peter Kraljic,^11^ the portfolio purchasing model classifies supplies (or supply categories) into four quadrants based on their financial and risk impacts on the business. Based on a supply item's classification, different procurement strategies are recommended. The model is a flagship in supply management and has undergone significant refinements and verification in subsequent research.^24^ A variation of the portfolio purchasing model was applied in this context to provide managerial insights about optimizing procurement.

## 3 METHODS

### 3.1 Research context and sample

We examined the cost of diabetes in Kuwait, a small Middle Eastern country with a high per capita GDP, where 18.8% of the adult population is diagnosed with diabetes.^27^ Data were collected from a comprehensive diabetes management outpatient facility in Kuwait that serves more than 3000 patients with diabetes per year. Data for 3538 patients were obtained. Patients with missing data or data‐entry errors for variables of interest were omitted, resulting in 2828 complete patient observations. The incomplete and erroneous records were compared to the rest of the sample to check for omission bias. When comparing patients with incomplete records (*n* = 710) with patients with complete records (*n* = 2828), no significant differences appeared between the two groups except that patients with incomplete records had significantly fewer clinic visits. Relevant information about the patients was retrieved without compromising their identities. Patient variables collected were age, gender, nationality, type of diabetes, clinic visits, blood sugar (HbA1c), and body mass index (BMI).

All dispensing transaction records between August 2017 and August 2018 were obtained and matched to patient records from the center's pharmacy department. The pharmacy processed an average of 76 patients per working day and dispensed 404 items daily. This data set included 98,648 records with information about the dispensed items, number of units dispensed, prescribing physician, pharmacist, affiliated patient, quantity, and time stamp.

The category, generic name, and dosage were listed for each item dispensed. As for pricing, we used a public directory published annually by Kuwait's Ministry of Health. In our analysis, we used wholesale prices.

### 3.2 Measuring the consistent of pharmaceutical prices across countries

To ensure that our findings are relevant and generalizable to other diabetes clinics (particularly in other countries), we collected medication pricing data from four other countries to examine their correlation with Kuwait's prices and calculate a price‐adjustment factor. We compared prices of the 60 most dispensed drugs in our sample, accounting for 90% of total expenditures, with prices from four other countries: the United States, the UK, Denmark, and Canada. High correlation coefficients indicate consistency in price‐setting across the different countries, supporting this study's generalizability. A price adjustment factor is calculated as the simple regression coefficient to indicate the difference in price magnitudes across countries.

### 3.3 Analysis

The dispensing data set was matched with the pharmaceuticals pricing data set to analyze supply consumption patterns in monetary costs and volume. The volume dispensed, total cost, and the number of patients receiving the drug were calculated for each pharmaceutical item. The results were aggregated at the clinical category and subcategory levels. Consumption and expenditures on items were calculated at the patient level to determine the total supply cost per patient per year. Additional variables included the number of unique drugs each patient received, pharmacy visits per patient, and the average number of items dispensed per patient visit. To fit the pharmaceutical items data onto the purchasing portfolio model, the total cost of the dispensed pharmaceutical item was used as a proxy for financial. A measure of product risk was used to quantify how many customers or products were exposed to the product. We considered the prescription prevalence—the percentage of patients who received at least one dose of the pharmaceutical item—as the proxy for risk. In other words, if a health warning or recall notice was issued for a pharmaceutical item, this measure reflects how many patients would need to be informed and have their prescription plans adjusted.

## 4 RESULTS

### 4.1 Measuring the consistent of pharmaceutical prices across countries

We searched for 60 common diabetes drugs in official databases of four countries (United States, UK, Denmark, and Canada) and recorded official pricing information for at least 38 exact matches in each database. The correlations between country price lists were calculated (Table 2). Diabetes medication prices were highly correlated across the sampled countries, supporting that even though prices may differ in absolute terms across these countries, differences in prices across drugs are generally consistent. The price adjustment factor in Table 2 captures the magnitude difference across price lists. For example, on average, drug prices in the United States were 4.4 times higher than Kuwait's prices. This is expected given the United States‘s exceptionally high cost of health care, which is demonstrated in Table 1. Denmark drugs were priced almost identically to Kuwait's prices, as indicated by the high correlation between the two price lists and a price adjustment factor close to 1.0.

**Table 2:** Cross‐country comparison of diabetes‐related drug prices

Country	*n* a	Price adj. factor^b^	Correlation matrix^c^				
			KUW	USA	DEN	UK	CAN
Kuwait	60	1.000	1				
United States	41	4.417	0.781	1			
Denmark	41	1.050	0.961	0.742	1		
United Kingdom	40	0.856	0.864	0.672	0.925	1	
Canada	38	0.767	.773	0.684	0.722	0.857	1

^a^ Sample size (*n*) is based on how many medications we could match precisely (by brand, dosage, form, and quantity per pack) from our reference list of 60 to formulary databases in other countries.

^b^ The price adjustment factor is a coefficient that estimates the magnitude difference in country prices.

^c^ Pearson correlation with pairwise deletion applied.

### 4.2 Patient and medication cost statistics

Descriptive statistics for the patients in the sample are presented in Table 3. Patients, on average, visited the pharmacy 5.26 times per year, receiving an average of 5.3 items per visit. The clinic treated about three times more patients with type 2 diabetes than type 1 diabetes, and the two groups differed significantly on most metrics, as seen in Table 3.

**Table 3:** Patient descriptive statistics

	Type I patients	Type II Patients
Observations	637	2191
Demographic statistics		
Gender (% male)	49.5	52.7
Average age	22.0 (11.6)	61.4 (11.4)
Average appointments attended	52.7 (30.8)	42.4 (32.2)
Average HbA1c	8.5 (1.6)	7.9 (1.5)
Average BMI	24.8 (5.5)	32.3 (6.6)
Per‐patient dispensing statistics		
Average annual pharmacy visits	3.8 (2.1)	6.0 (2.9)
Average number of prescribed items	4.7 (3.15)	11.9 (2.9)
Average annual medication costs (EUR)	592 (476)	1418 (1,050)

*Note*: *N*= 2828; values represent means and standard deviations (in parentheses) unless otherwise noted.

On average, 9.9 products were prescribed to each patient, with a median of 9 and a standard deviation of 6.5. Patients with type 2 diabetes required more pharmaceutical supplies in terms of variety and volume; 11.9 items compared with 4.7 for patients with type 1 diabetes. Even after controlling for demographic factors such as age and BMI, patients with type 2 diabetes received, on average, 2.7 more medications. Consequently, the average pharmaceutical cost for these patients was significantly higher than those with type 1 diabetes.

The overall average cost for medications per patient was 1231 Euros (all monetary values were converted from the local currency of Kuwaiti dinars to Euros at a 0.358 currency exchange rate observed on 20 April 2020). Patients with type 2 diabetes exhibited higher costs but differed significantly on other demographic variables such as average age and BMI. Based on a regression analysis, patient age, BMI, HbA1c level, and the number of clinic visits were all positively and significantly correlated with medication cost (Appendix A). Gender, however, was not significantly associated with medication cost or the number of medications dispensed. On average, a 10‐year age difference increased the medicines prescribed by three. Every 10 additional BMI units above the average were associated with 1.2 additional prescriptions, which translates to an additional €265 in annual pharmaceutical costs per patient.

The 287 unique products dispensed during the study period were classified into 20 clinical categories and 64 subcategories. Some drugs were available in multiple doses and in varied packaging; we counted each stock‐keeping unit as a different item. Therefore, a drug like Glucophage, which comes in six dose varieties, was calculated as six unique items. The four largest categories were antidiabetes, insulin, anti‐lipidemic, and anti‐hypertensive drugs (Table 4). Collectively, these categories accounted for 59% of drugs dispensed by volume and 81% by cost. The most dispensed item was a vitamin D3 supplement, followed by insulin pens. In terms of the total cost (volume multiplied by the price per unit), the Lantus SoloStar insulin pen exhibited the highest portion of supply expenditures.

**Table 4:** Pharmaceutical item categories

Item category	Cost (% of total pharmaceutical expenses)	Volume^a^ (% of total items dispensed)	Prescription prevalence^b^ (%)
Antidiabetes medications	29.3	21.3	66.2
Insulin	28.0	12.6	62.9
Anti‐lipidemic medications	15.9	11.1	67.1
Antihypertensive meds	8.0	13.7	53.7
Neurology medications	4.1	3.6	20.9
Vitamins and minerals	3.0	9.4	63.0
Cardiology medications	2.4	5.2	31.9
All other categories	9.2	23.1	‐‐

^a^ Data were aggregated from 98,648 transactions. Each stock‐keeping unit was considered a unique item.

^b^ Percentage of patients (out of the sample of 3538 patients) who received at least one item from the category during the study period (September 2017–August 2018).

**Figure 2:**
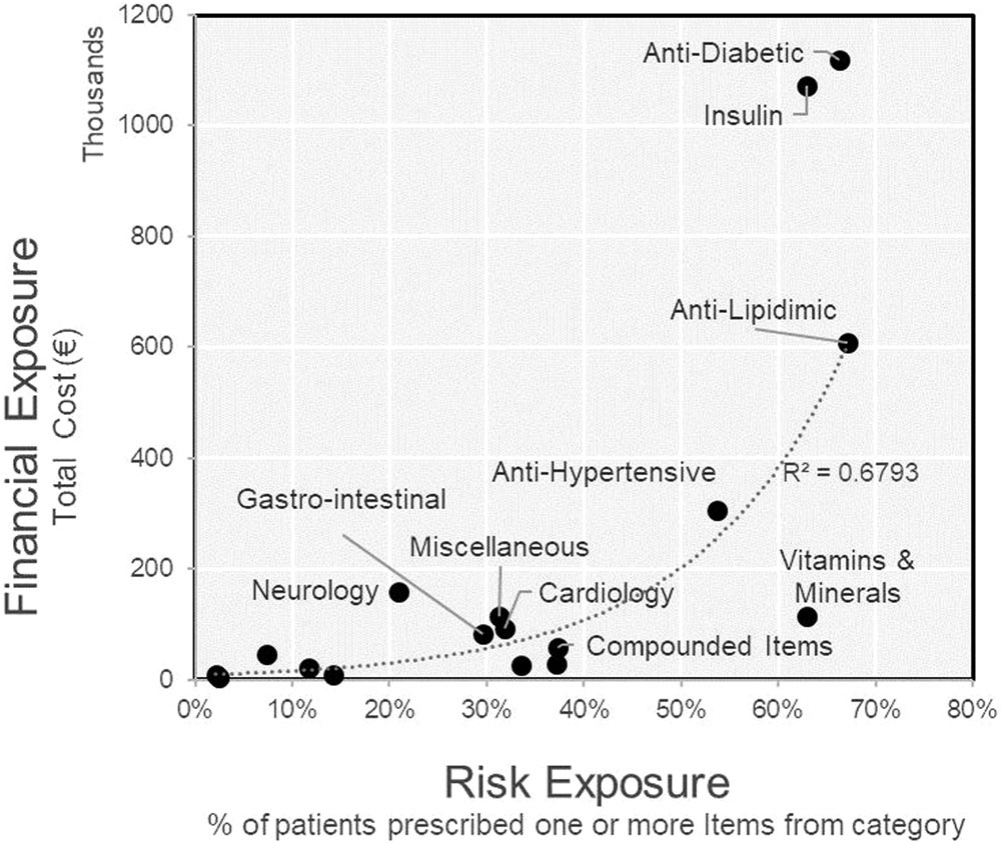
Supply classification matrix for pharmaceutical categories related to diabetes treatment.

**Figure 3:**
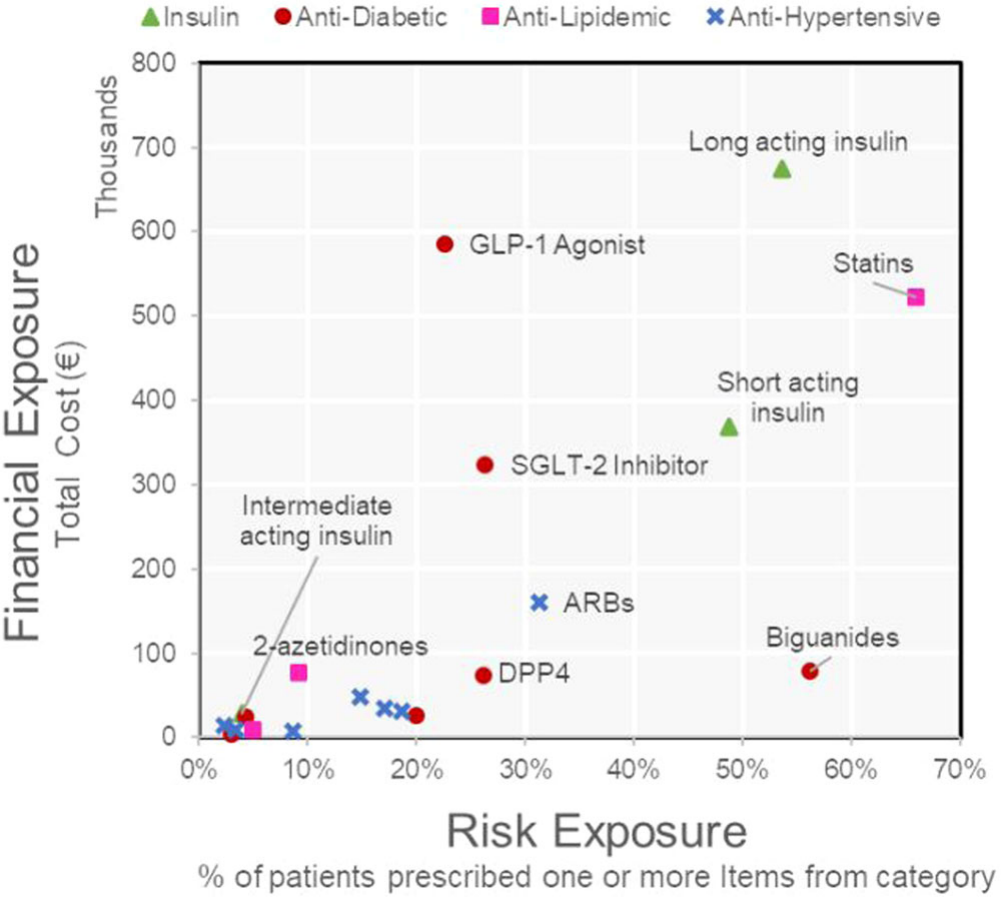
Supply classification matrix for pharmaceutical subcategories related to diabetes treatment.

The scatter plot presented in Figure 2 shows the total cost of the category against the percentage of patients at the center who received items from this category. Figure 3 applies the same concept at the subcategory level. The subcategories of the four largest categories (regarding financial impact and volume dispensed) were plotted. The results in Figure 3 indicate that items in the same drug category may diverge from a procurement perspective.

## 5 DISCUSSION

### 5.1 Classifying supplies used in diabetes management

Understanding the characteristics and costs of supplies enables health administrators to identify the primary sources of costs and their drivers to improve procurement efficiency. Projecting the data onto a standard purchasing portfolio model (Figure 1) provided interesting insights. The costliest and most dispensed supply categories were antidiabetes and insulin products, appearing in the upper‐right quadrant of Figure 2. These can be classified as strategic supply items and require the most attention in forecasting, supplier relations, risk assessment, logistics, and inventory management. Anti‐lipidemic and anti‐hypertension drugs, which address high cholesterol and high blood pressure (two ailments highly associated with obesity), followed in supply expenditures.

An interesting drug category was the vitamins and minerals category, appearing in the bottom‐right quadrant in Figure 2. This category was among the top five highest in terms of patient exposure. Vitamin D3 was the most dispensed item in our study. In our sample, more than 50% of patients received a vitamin D prescription. Vitamin D deficiency is a global public health issue, particularly in the Middle Eastern region where this study was conducted.^28^ Recent clinical research also identified a possible association between vitamin D deficiency and diabetes.^29^ From a supply management perspective, vitamins and minerals can be classified as bottleneck items, which broadly impact customers but exhibit relatively low financial impact on organizations. With bottleneck items, exploiting full buyer power is encouraged: ensuring the availability of products, identifying product substitutes and innovations, and ensuring safety stocks.

Most drug categories appeared in the lower‐left quadrant of Figure 2, representing noncritical items. It is important to note that this is not a reflection of the clinical importance of these items to patient health. Instead, from a procurement perspective, such items require a small portion of the budget and have a lower impact on the overall patient population, should there be changes or a shortage in supply. To gain price and logistics efficiency, strategies for dealing with such items include automating reorders, considering vendor‐managed inventory agreements, and establishing blanket contracts for multiple products in this category.

Interesting insights emerged when implementing the supply classification scheme at the subcategory level (Figure 3). In many cases, subcategories of drugs belonging to the same category diverged in classification. For example, the three subcategories of insulin (long‐acting insulin, intermediate‐acting insulin, and short‐acting insulin) each occupied different regions of the scatterplot (Figure 3) but remained on the general trendline. Two antidiabetes drug subcategories, biguanides and GLP‐1 agonists, varied in supply classification and deviated significantly from the trendline. Biguanides were characterized as bottleneck items, whereas GLP‐1 agonists were deep in the leverage items quadrant. With leverage items, exploiting full buyer power is encouraged: vendor selection, product substitution, multisourcing, and order volume optimization. Another subcategory of anti‐diabetes drugs, DDP4, was in the class of noncritical items, given their low financial impact and relatively low prescription prevalence.

Unaware materials managers may be inclined to source clinically similar items from the same supplier with identical contract terms. However, examining these supplies’ financial and risk factors suggests that each subcategory should receive a different level of consideration. This analysis also can be conducted at the product level to inform hospital administrators about items that require more strategic attention to improve overall procurement effectiveness.

Finally, comparing the drug prices in this study's context to prices of the same products in other countries is important to establish the generalizability of this study. In many costing studies, the results may not apply to other contexts due to significantly different cost structures across different healthcare facilities. The statistics comparing prices of the top 60 diabetes medications across four representative countries (Table 2) provide evidence that cost estimates and analysis can be generalized to other contexts. The United States’ drug costs appeared systematically higher in magnitude than other countries, likely due to the different health reimbursement structures. Manufacturers may price drugs differently in different countries because of logistics costs, tariffs, regulatory factors, or other reasons. Investigating pharmaceutical drug price differences across countries is a rich area of study in health economics.

### 5.2 Limitations and future research opportunities

Issues in the data presented minor limitations worth mentioning. First, approximately 20% of patient records were omitted due to incomplete or erroneous data from the population of patients who visited the clinic. Although we analyzed the missing data using what was available in those records and found minimal bias risk, there is no way to eliminate such risk. Furthermore, the dispensing data from the pharmacy department covered 1 year, with an arbitrary starting date. Selecting an extended period or one with a different starting date may have included or excluded other patients and line items, thus altering the results. However, we believe this risk is low because our selected period captured more than 90,000 dispensing transactions, and most patients in the sample had multiple dispensing records. Third, our sample featured patients who visited a treatment center in Kuwait. Therefore, biases may exist in this sample selection. First, patients who receive treatment at this center may present a different level of diabetes severity than the general population, which requires them to visit this specialized clinic. Some patients with diabetes receive treatment and medications at primary care clinics. This issue raises an interesting research question: Is a specialized diabetes care center more clinically or financially effective in serving patients with diabetes than a primary care health center? Furthermore, we did not track whether patients acquired additional supplies from other sources. Nonetheless, the clinic in the study is meant to provide all necessary services and supplies to patients. Therefore, we can presume that additional spending on supplies by patients was minimal.

Although prices differed in magnitude across countries, they were highly correlated. Therefore, supply costs associated with diabetes treatment in Kuwait are comparable with those of other countries after applying a price adjustment. This can begin to illustrate the impact of certain socioeconomic and policy factors on the economic burden of diabetes on nations. Is there an economies‐of‐scale effect whereby countries with a higher prevalence of diabetes, such as Kuwait, learn to serve their populations more cost‐effectively, controlling for other factors? This study's specialized diabetes research and treatment center was a direct response to the relatively high prevalence of diabetes in the country.

### 5.3 Implications

This study scrutinized pharmaceutical supplies provided to patients with diabetes at a specialized diabetes clinic in Kuwait. The results of this study provide both health policy implications for the country's health system and managerial implications for clinics that treat people with diabetes. Pharmacists and hospital administrators can apply insights from the supply cost analysis to improve their purchasing and dispensing practices. In addition to clinical factors, understanding the business risks and financial factors related to pharmaceutical categories and specific products can steer managerial decision‐making toward more efficient supply management. This study's relatively straightforward portfolio purchasing model is a powerful tool for prioritizing procurement activities. This study found that even related products (such as different types of insulin) may require markedly different approaches for an optimal sourcing strategy. Such investigations at the item level may also reveal opportunities for product standardization, generic drug substitution, automated procurement, or vendor‐managed inventory policies.

Ample opportunities remain for further analyzing pharmaceutical costs and consumption patterns for patients with diabetes. Our findings provide benchmarks regarding pharmaceutical expenses and product consumption patterns for people with diabetes. With some adjustment factors, the results of this study can be generalized and compared with results from patients with diabetes receiving treatment at other clinics. This study may serve as a model for examining the costs of other supply‐intensive diseases in the population, such as cardiology, oncology, and orthopedics. Although ample clinical studies considered the economic burden of diabetes, this study examined the cost of supplies through a procurement frame of reference. This perspective provided valuable insights for both health policy and health administration.

## AUTHOR CONTRIBUTIONS

**Yousef Abdulsalam**: Conceptualization; data curation; formal analysis; investigation; methodology; project administration; writing—original draft; writing—review & editing. **Abdullah Alibrahim**: Data curation; formal analysis; investigation; methodology; supervision; writing—original draft; writing—review & editing. **Dari Alhuwail**: Conceptualization; data curation; formal analysis; methodology; project administration; validation; writing—review & editing. **Hashem Behbehani**: Methodology; project administration; resources; validation; writing—review & editing.

## CONFLICTS OF INTEREST

The authors declare no conflicts of interest.

## TRANSPARENCY STATEMENT

The lead author, Yousef Abdulsalam, affirms that this manuscript is an honest, accurate, and transparent account of the study being reported; that no important aspects of the study have been omitted; and that any discrepancies from the study as planned (and, if relevant, registered) have been explained. All authors have read and approved the final version of the manuscript. Yousef Abdulsalam had full access to all the data in this study and took complete responsibility for the data's integrity and the data analysis's accuracy.

## Data Availability

Yousef Abdulsalam had full access to all the data in this study and took complete responsibility for the data's integrity and the data analysis's accuracy.

## APPENDIX A PREDICTORS OF ANNUAL PHARMACEUTICAL COSTS PER PATIENT

**Table:**

	Dependent variable: Annual pharmaceutical costs per patient
Intercept	290.02*** (18.84)
Gender (1 = male, 0 = female)	5.22 (11.57)
Age	1.74*** (0.50)
Nationality (1 = citizen, 0 = expatriate)	183.16*** (17.99)
Clinic visits	1.88*** (0.18)
Diabetes type (1 = type 1, 0 = type 2)	−169.26*** (24.56)
BMI (most recent)	9.40*** (0.88)
HbA1c (most recent)	22.20*** (3.79)
Observations	2,828
*R* ^2^	0.233
Residual SE	300.71 (*df* = 2,820)
F‐statistic	122.16*** (*df* = 7, 2,820)

*Note*: **p* < 0.10. ***p* < 0.05. ****p* < 0.01. Continuous variables were centered.
